# Revealing gaps in marine evidence with a natural capital lens

**DOI:** 10.1098/rstb.2023.0214

**Published:** 2025-01-09

**Authors:** A. J. Fairbrass, K. Fradera, R. Shucksmith, L. Greenhill, T. Acott, P. Ekins

**Affiliations:** ^1^ University College London Institute for Sustainable Resources, Central House, 14 Upper Woburn Place, London WC1H 0NN, UK; ^2^ University of the Highlands & Islands Shetland, Gremista, Lerwick, Shetland ZE1 0PX, UK; ^3^ Howell Marine Consulting, 23 Hauxley Links, Low Hauxley, Morpeth NE65 0JR, UK; ^4^ University of Greenwich, Old Royal Naval College, Park Row, London SE10 9LS, UK

**Keywords:** marine natural capital, ecosystem services, marine decision-making, evidence gaps, coastal and marine ecosystems, UK marine environmental policy

## Abstract

The natural capital concept positions the natural environment as an asset, crucial for the flow of goods and benefits to humanity. There is a growing trend in applying this concept in marine environmental management in the United Kingdom (UK). This study evaluates six varied marine decisions across England, Scotland and Wales. It focuses on the evidence informing these decisions and the extent to which they represent the complete spectrum of marine natural assets and ecosystem services. We identified a reliance on various evidence types, including consultations, data and statistics, maps and literature reviews. Natural assets such as aquatic resources and energy sources were most frequently evidenced. Fishing was the predominant provisioning ecosystem service benefit. There was a notable gap in evidence on marine habitats’ water quality regulation service. Recreation and tourism dominated the cultural ecosystem service evidence, with less focus on indirect uses such as spiritual nature connections. We reveal gaps in the evidence in marine decisions on significant marine ecosystem service benefits. Our study provides additional evidence to an already identified need to fill evidence gaps in marine water regulation and non-use values of the UK’s marine environments.

This article is part of the discussion meeting issue ‘Bending the curve towards nature recovery: building on Georgina Mace's legacy for a biodiverse future’.

## 1. Introduction

Ecosystems provide multiple services essential for human well-being, including provisioning, regulating and cultural services [1]. Traditional environmental management approaches, focused on maximizing yields through equilibrium-based methods, are insufficient for addressing ecosystems’ dynamic and unpredictable nature, presenting a ‘wicked problem’ in management [2]. Sector-specific administrative structures often result in siloed decision-making, limiting the consideration of multifaceted ecosystem services in management decisions. Natural capital approaches, which advocate for a holistic view of ecosystem services, offer a promising solution [3,4] but are hindered by gaps in evidence about natural capital [5]. This paper aims to pinpoint these evidence gaps and analyse how well the evidence used in marine decisions in the United Kingdom (UK) reflects the multiple values of natural capital and ecosystem services.

### (a) Overview of the article structure

This article is organized into several sections to provide a comprehensive and coherent overview of the types of evidence used in marine decisions, the representation of marine natural capital and ecosystem services values and identifying evidence gaps. Section 2 details the methodological approach employed in the study, outlining the process of selecting representative case studies of marine management decisions from England, Scotland and Wales and the subsequent review of relevant literature to identify the types of evidence used, the components of natural capital represented and the measures of natural capital in the evidence base. Section 3 presents the types of evidence utilized in these case studies and the representation of natural capital components and identifies significant gaps in the evidence. Section 4 delves into the discussion of the results, focusing on the identification of research gaps and the implications of these gaps for marine decisions. Finally, the study’s limitations and directions for future research are discussed, leading to a conclusion reiterating the implications of the study’s findings.

### (b) Literature review

Coastal and marine ecosystems in the UK play a crucial role in supporting biodiversity and providing essential goods and services to humans [6,7]. These ecosystems encompass a variety of habitats, including seagrass beds, salt marshes and estuaries, which are home to numerous species of plants and animals [8–11]. In addition to their ecological significance, coastal and marine ecosystems contribute significantly to the UK economy through activities such as tourism, fisheries and renewable energy production [12,13]. However, these ecosystems face many challenges from anthropogenic pressures, including pollution, climate change, invasive species, overfishing and damaging fishing practices such as bottom trawling and the development of infrastructure for various uses including offshore wind, oil and gas extraction and carbon capture and storage [8–11,14]. These pressures jeopardize the ecological integrity of coastal and marine ecosystems and undermine their ability to provide vital ecosystem services and support human health and well-being [15]. Therefore, there is an urgent need for effective and informed management of these ecosystems to ensure their long-term health, resilience and sustainability.

Natural capital refers to the stock of environmental assets that supports a flow of ecosystem services that benefit society [3]. In the marine environment, this includes living and non-living marine resources, such as seabed/marine benthic habitats, the water column and cultivated and naturally occurring aquatic organisms, such as fish and crustaceans, that provide a range of ecosystem services benefits to humans [16]. The Millenium Ecosystem Assessment classified ecosystem services as provisioning, regulating, supporting and cultural [1]. In the marine environment, provisioning services include the production of goods such as seafood, raw materials and genetic resources, while regulating services encompass climate regulation, waste processing and coastal protection [17]. Supporting services, like nutrient cycling and habitat to support biodiversity, underpin the functioning of ecosystems and enable the delivery of other services [1,18]. Finally, cultural services represent the non-material benefits of marine ecosystems, including recreation, aesthetic enjoyment and cultural heritage [19]. The sustainable management of marine natural capital is critical to ensure the continued flow of these goods and benefits, which significantly impact human health and well-being [20]. These services mitigate coastal flood risks, support jobs and livelihoods through well-managed fish stocks and reduce the need for costly engineering solutions for flood mitigation and carbon sequestration. Effectively managing these ecosystem services is crucial for economic development and maintaining resilient coastal communities.

In the UK, specific marine management organizations are adopting the concept of natural capital to improve their approach to marine management and decision-making. This approach moves from a narrow focus on individual components, such as fishing, recreation or offshore wind energy, towards a more holistic perspective, encouraging viewing the ocean as a comprehensive, interconnected system. The UK’s Department for Environment, Food and Rural Affairs (DEFRA) has adopted the natural capital approach in its 25 Year Environment Plan [21]. DEFRA is delivering the Natural Capital and Ecosystem Assessment Programme (NCEA), a 3-year multi-million-pound Treasury-funded science programme [22]. The programme’s main aims are to change how the government makes environmental decisions using a natural capital approach and to fill evidence gaps about natural capital. The Scottish Government uses the ecosystem services language in its National Marine Plan [23] and the natural capital language in its Vision for Scotland’s Blue Economy [24] and has developed a marine natural capital asset index [25]. The Marine Management Organisation (MMO) is exploring the development of a five capital approach—which includes natural capital, for marine planning [26]—and The Crown Estate, which owns and manages the seabed around the UK, is exploring the development of financial markets for marine natural capital [27]. The Office for National Statistics (ONS) has developed an experimental marine natural capital account for the UK [28,29], while natural capital approaches have been applied to inform marine management decisions in several fisheries in UK waters [30].

Natural capital approaches have attracted criticism owing to their anthropocentric framing of nature, inability to include certain cultural values and the involvement of economic valuation, which it is feared will lead to the commodification of nature [31–34]. Bateman *et al*& Mace [4] argue that the concept is often misunderstood as being narrowly focused on the economic valuation of nature. Mace [3] clarifies that natural capital approaches should

*‘not be simply a means to place a monetary value on the natural environment so that it is taken more seriously in decision-making. Rather, [they] should be the means by which governments, corporations, and individuals can take proper responsibility for the essential components of natural capital that underpin society and a good life (the assets), record the condition of these assets and how this is changing over time, and ensure that provision is made when they fall below critical levels’*. [p. 55]

Natural capital approaches do not simply consider the monetary valuation of nature and the benefits we derive from it, but instead use a structured approach for comprehensively understanding nature and our dependence on it in biophysical, social and economic terms.

Evidence plays a pivotal role in guiding regulators towards informed outcomes in marine decision-making within the UK. Consideration of specific receptors and factors in these decisions is influenced mainly by existing legislative frameworks, such as the Marine and Coastal Access Act (2009) [35]. Additionally, planning processes require such factors to be relevant, measurable and reasonable to satisfy the materiality principle in order to be considered in decision-making [36]. This approach ensures that decisions are grounded in significant and appropriate evidence, in line with legal and environmental goals. It implies that with respect to natural capital, certain ecosystem service benefits like heritage preservation may be deemed material and thus influential in marine planning. In contrast, others, like the viewing of seascapes, might not meet these criteria and are therefore not prioritized.

Various types of information influence marine decisions, such as environmental research, economic data and sociocultural information [37,38]. Environmental data are critical in shaping policy and decision-making, including species distributions, habitat quality, ecological interactions and environmental trends [39]. Economic data, such as resource values, market analyses and cost–benefit assessments, help to evaluate the trade-offs between different policy alternatives [40,41]. Finally, sociocultural information, including local knowledge and cultural values, provides insights into the societal implications of marine decisions and helps to ensure that policies are sensitive to local contexts and social equity concerns [42,43].

There is a growing recognition of the need to understand the types of evidence and values that inform marine decisions and to identify potential gaps in our understanding of marine natural capital and its associated benefits [44,45]. A comprehensive review of the evidence used in UK marine decisions is part of ensuring that decisions are transparent, robust and based on the best available information [46]. Furthermore, in the face of increasing pressures on marine ecosystems, such as climate change, pollution and overexploitation of resources, it is crucial to identify and fill any evidence gaps that might hinder the achievement of statutory marine duties [47–49]. The primary objectives of this research are therefore to examine how evidence is used in marine decision-making in practice and to evaluate this against the key components represented in natural capital and ecosystem service frameworks.

This study aims to address the following research questions: (i) what types of evidence are utilized in UK marine decisions? (ii) How representative is this evidence of the key components of natural capital and ecosystem services in the marine environment? (iii) Are there any significant gaps in the representation of the key components of natural capital and ecosystem services in the evidence base? This study aims to provide valuable insights for policymakers and stakeholders as they seek to develop the natural capital approach to marine management in the UK, by investigating evidence related to natural capital and ecosystem services. It has significant implications for the understanding of evidence used in marine decisions. We do not aim to assess the suitability of the concepts of natural capital and ecosystem services for marine environmental management. Instead, our analysis compares the practical evidence with two specific natural capital and ecosystem service frameworks to assess whether it aligns with the key components within those frameworks.

This study provides a comprehensive assessment of the types of evidence and the values of natural capital and ecosystem services represented in a diverse array of marine decisions across England, Scotland and Wales. A robust and systematic approach was employed to address the research questions, which involved a comprehensive review of the publicly available documents supporting six representative marine decisions from England, Scotland and Wales between 2019 and 2023. The overarching objectives of the wider UK-government-funded Diverse Marine Values project [50] guided the selection of the six marine decisions for review. The Diverse Marine Values project focuses on three test sites: Portsmouth, England, the Upper Severn Estuary, bordering both Wales and England, and the Shetland Islands in Scotland. These test sites collectively represent a range of UK marine and terrestrial geographies, nationally and locally tailored governance systems, diverse communities and distinctive cultural identities. We selected two case studies of decisions at different scales (national, regional or local) for each test site. Case studies were chosen based on their relevance to specific themes at each test site: coastal defence and access in Portsmouth, water quality in the Upper Severn Estuary and rural growth/transition in Shetland.

We thoroughly examined the relevant documents to identify the types of evidence used in these case studies, such as expert consultation, raw data and statistics from *in situ* environmental surveys. The analysis also focused on the components of natural capital represented by the evidence, including ecosystem and commodity assets and the flow and benefits from ecosystem services. The measures of natural capital represented by the evidence were also scrutinized, such as the biophysical flows of ecosystem services and the economic value of goods and benefits derived from natural capital. The start of the decision-making processes that led to our reviewed decisions pre-dates the appearance of the natural capital concept in the UK marine management landscape. Therefore, we did not assess how well the natural capital concept was applied in the decisions, but instead, we conducted a retrospective assessment of how well natural capital and ecosystem service values were represented.

## 2. Methods

### (a) Overview of the case studies

The six case studies that were selected for review represent various marine policy and management contexts across England, Scotland and Wales [51–55]. We chose these case studies to provide a comprehensive understanding of the types of evidence used and values of natural capital and ecosystem services represented in various marine decisions. We define a decision as the endpoint of the case study—the point at which a material determination is made. Related to this, the term 'decision-making process' provides a temporal boundary to the analysis. It defines the start of the process under consideration, which was chosen based on professional judgement. We selected case studies that have completed a decision-making cycle rather than being still in progress to ensure that the complete set of publications associated with the case study were available for review. This approach allowed for a comprehensive examination of marine decisions across various scales and contexts, providing valuable insights into the types of evidence used and values of natural capital and ecosystem services considered in marine policy and management.

#### (i) Sectoral Marine Plan for Offshore Wind Energy (Scotland), 2020

Development of the sectoral plan for offshore wind energy in Scotland culminated in a decision on the spatial distribution of leasing areas for offshore wind development. This is critical to Scotland’s efforts to transition towards renewable energy sources and achieve its climate change mitigation goals. It provided a strategic assessment for the ScotWind leasing round of offshore wind energy projects in Scotland’s marine environment.

#### (ii) Sullom Voe Harbour Masterplan, Shetland, 2022

The Sullom Voe Harbour Masterplan aims to establish a strategic framework for the sustainable growth and development of the Sullom Voe Harbour area in Shetland. The decision was the adoption of a development plan for the Sullom Voe Harbour. The Masterplan is significant because it seeks to balance economic growth, environmental protection and social well-being in the context of port and industrial development while considering the region’s unique marine and coastal ecosystems.

#### (iii) Southsea Coastal Defence Scheme, 2021

The Southsea Coastal Defence Scheme aims to enhance coastal protection measures in Southsea, England. The scheme addresses the increasing risks of coastal erosion and flooding owing to climate change, and its implementation is essential for safeguarding the region’s local community, infrastructure and valuable coastal ecosystems. The decision involved granting of the marine licence and planning permission for the scheme as well as an Environmental Impact Assessment (EIA) consent decision.

#### (iv) Southsea Coastal Defence Scheme sub-frontage 4, 2023

This decision focuses on the approval of a specific sub-frontage within the broader Southsea Coastal Defence Development. It differs from the decision to approve the wider scheme in being much smaller-scale and employing different consultation approaches.

#### (v) Welsh National Marine Plan (WNMP), 2019

The WNMP is a comprehensive policy framework guiding Wales' sustainable development and management of marine and coastal resources. This Plan sets the strategic direction for various sectors operating within the marine environment, including renewable energy, fisheries and aquaculture. It strives to balance the competing demands on the marine environment while safeguarding its ecological integrity. The decision was the approval of the Plan.

#### (vi) River Wye (Wales and England) and River Usk rod and line (salmon and sea trout) byelaws, 2021

This case study pertains to the joint renewal of three fishing byelaws for the Wye and Usk Rivers in Wales, including the River Usk Byelaws, the River Wye Byelaw for England and the River Wye Byelaw for Wales (hereafter referred to as the Rivers Wye and Usk Byelaw) [56]. It aims to regulate fishing activities and ensure the sustainable management of fish stocks while considering the broader implications for the health and resilience of the rivers’ ecosystems and their associated services. The decision was the adoption of the renewed byelaw.

### (b) Document review and data collection

The document review and data collection process involved gathering relevant documents and sources for the six case studies. We compiled various materials, such as consultation reports and consultee comments, sustainability assessments, socio-economic assessments, HRAs, Environmental Statements, inquiry reports and other relevant documents. The documents reviewed are listed in the electronic supplementary material. All documents reviewed were publicly available online at the time of the review.

### (c) Data analysis using NVivo

#### (i) Coding of types of evidence

The data analysis process involved using NVivo software [57] to systematically code the various types of evidence identified in the collected documents. The coding process began with classifying different types of evidence, such as expert consultation, raw data and statistics from *in situ* environmental surveys. To ensure a robust and well-structured coding framework, we based our typology of evidence on the Informational Pyramid presented in the United Nations (UN) Statistics Division’s ‘System of Environmental–Economic Accounting (SEEA) Applications and Extensions’ paper [58]. The SEEA is an international standard that offers a structured approach to natural capital accounting. Its alignment with government information structures makes it a relevant and credible starting point to investigate the evidence used in government decision-making. Moreover, we successfully applied it in previous work reviewing natural capital information use in policymaking [59]. This framework served as a starting point for the coding process, allowing us to identify and categorize the different forms of evidence found in the documents. The analysis incorporated additional classes of information when necessary, expanding and refining the initial framework. The typology of evidence types included in the coding were as follows:

#### Assessment tools

These integrated evaluations combine various data sources under a structured framework to assess specific topics. *Example*: a comprehensive coastal process assessment was conducted to evaluate the impact of development on erosion, included in the EIA for the Southsea Coastal Defence Scheme.

#### Case studies

Detailed examinations of specific instances to illustrate broader phenomena or trends. *Example*: the energy potential of tidal turbine deployment in the Bristol Channel, highlighting renewable energy opportunities.

#### Consultation

Gathering opinions and feedback from stakeholders and the public. *Example*: public and online consultations were conducted to gather community input on the Southsea Coastal Defence Scheme’s design [60].

#### Data and statistics

This involves collecting raw quantitative or qualitative data and summarizing it using statistical analyses. *Example:* the Southsea Coastal Defence Scheme’s Environmental Statement uses fish monitoring data from Southampton Water to assess ecological impacts.

#### Databases of evidence

Compilations of data from multiple sources organized in a searchable format. *Example*: the Joint Nature Conservation Committee’s (JNCC) Marine activities and pressures evidence database informs the Habitats Regulation Assessment (HRA) of the WNMP[61] .

#### Environmental standards

Set benchmarks for acceptable levels of environmental quality. *Example*: water quality standards from the Water Framework Directive, cited in Wales’ Marine Evidence Report [62] to evaluate chemical parameters.

#### Expert judgement

Insights from individuals recognized as authorities in their field. *Example*: insights from a Southern Inshore Fisheries and Conservation Authority member on Solent fishing activities, cited in the Southsea Coastal Defence Scheme Environmental Statement [63].

#### Feasibility studies

Evaluations of the practicality and potential outcomes of proposed projects. *Example*: an analysis of the Severn Estuary’s tidal power industry potential, informing the Wales’ Marine Evidence Report [62].

#### Good practice guidelines

Recommendations on best practices in specific areas. *Example*: JNCC protocols for minimizing ecological risks from marine construction, referenced in the WNMP’s HRA [64].

#### Government responses

Governmental feedback to reports, inquiries or public input. *Example*: a Welsh Government response to a food security report, cited in the Wales’ Marine Evidence Report.

#### Indicators

Single metrics are used to represent specific phenomena or trends. *Example*: Scotland’s Index of Multiple Deprivation indicating coastal communities’ social characteristics in regional locational guidance for offshore wind energy [65].

#### Interviews, questionnaires and workshops

Primary data are collected directly from individuals or groups. *Example*: a study on the Welsh population’s values for the sea, derived from interviews cited in the Wales’ Marine Evidence Report [66].

#### Literature reviews and evidence syntheses

Comprehensive analyses of existing research, integrating findings from multiple studies. *Example*: a synthesis of managerial perspectives on catch-and-release fishing, offering insights for the Rivers Wye and Usk Byelaw public inquiry [67].

#### Maps

Visual representations showing the geographical distribution of various phenomena. *Example*: development zone maps for the Sullom Voe Harbour area, supporting spatial planning discussions [51].

#### Modelling outputs

Outputs produced from statistical modelling analyses. *Example:* predicted areas of seabed habitats cited in the Scottish Sectoral Marine Plan for Offshore Wind Energy [68].

#### Photos

Photographs are used for visual illustration. *Example:* underwater photographs used to illustrate the condition of a seabed habitat in the Environmental Statement of the Southsea Coastal Defence Scheme.

In our methodology, we acknowledged the interconnectedness between different types of evidence. For instance, while modelling outputs can be depicted in maps, it is crucial to recognize that not all models are reported in this way, and maps can derive from various sources beyond modelling outputs. Consultations may incorporate interviews, questionnaires and workshops, but these tools do not solely constitute consultation processes and consultations can employ other methodologies. Our coding approach was grounded in the presentation of evidence within the reviewed documents. Consequently, a map in the reviewed documents was coded as a map, even if it displayed model results, prioritizing how evidence was directly represented over its underlying methodology or source.

#### (ii) Coding of components of natural capital

The data analysis further involved using NVivo software to code the components of natural capital represented by the evidence in the reviewed documents. This process followed the UN SEEA classification system for natural assets and the Common International Classification of Ecosystem Services (CICES) [69] for ecosystem services. These frameworks have been adopted by the ONS Natural Capital Accounts [70].

These classification systems offer a structured framework but have yet to develop for fully marine and coastal environments. Therefore, to adequately capture the range of natural capital components and ecosystem services specific to the case studies, the SEEA and CICES frameworks were supplemented with additional classes of marine and coastal natural assets and ecosystem services from the Global Ocean Accounts Partnership (GOAP) [16]. We excluded from our analysis several ecosystem services present in the CICES and GOAP classification systems because they are not relevant to the UK marine context. This included genetic material use because no development would be of sufficient scale for this to be pertinent to decision-making processes. However, gene pool maintenance—a regulation and maintenance ecosystem service—was included. Other excluded ecosystem services were pest regulation, smell reduction and visual screening.

The decision-making processes reviewed in this study pre-date the adoption of the natural capital concept within the UK marine management landscape. Consequently, natural capital terminology was rarely encountered in the documents. To ensure comprehensive identification of natural capital components, we did not restrict our analysis to evidence explicitly described using natural capital language. Instead, the coding was conducted by a natural capital expert (A.J.F.), who applied their expertise to interpret and identify relevant evidence. This approach allowed us to translate descriptions into the classification systems of the SEEA and CICES frameworks, ensuring that relevant evidence was captured even when it was not explicitly framed in natural capital terms. This comprehensive and tailored approach ensured that the coding process accurately reflected the diverse range of marine and coastal natural capital components and ecosystem services in the documents. Furthermore, by utilizing and supplementing established classification systems, the analysis could effectively capture the full breadth of natural capital components informing UK marine decisions.

#### (iii) Coding of measures of natural capital

The coding of measures of natural capital represented by the evidence was a vital component of the data analysis process. This coding adhered to the structured approach provided by the Natural Capital Indicators Framework (NCIF) proposed by Fairbrass *et al*. [71]. The NCIF advocates for a comprehensive suite of measures that capture various aspects of natural capital. These measures encompass the quantity, quality and value of natural assets; biophysical measures of flows of ecosystem services; and social and economic measures of the goods and benefits derived from ecosystem services. In addition, the framework also includes measures for the economic and human inputs required to derive goods and benefits from natural assets, as well as economic, environmental and social measures of the residuals produced through the process of deriving goods and benefits from natural assets.

#### (iv) Comparative analysis of marine case studies

Following the coding process in NVivo, we leveraged the resultant data to conduct a comparative analysis of the evidence utilized in each of the six case studies. This analysis involved contrasting and comparing the types of evidence, the components of natural capital represented and the measures of natural capital utilized in each case study.

The NVivo software offers three metrics for analysing coded data: the number of words coded, the number of files coded and the frequency of unique sections of text coded. We used the frequency of unique text sections coded for our analysis because it most accurately reflects the number of distinct references to evidence within the documents reviewed. We chose this metric over others for several reasons. First, the number of words coded can vary significantly based on the extent of the discussion, potentially skewing results. Second, the number of files coded was not used because a single file could contain multiple citations of different evidence sources, which this metric would underrepresent. By focusing on unique text sections, we better approximated the number of evidence sources cited in the case studies. We assessed the frequency of evidence types cited across four natural capital components (natural assets, provisioning, cultural and regulation and maintenance ecosystem services). Utilizing NVivo’s Matrix Coding Query, we created a matrix displaying intersections (text that is coded at two different codes) between evidence types and natural capital components, quantifying co-occurrences of coding. Summary statistics, including the minimum, maximum, median and interquartile ranges, were calculated for these intersections. Based on these statistics, we categorized the frequency of intersections into four levels: low, medium, high and very high. This approach allowed us to identify patterns in evidence types associated with different natural capital components, revealing specific evidence preferences.

## 3. Results

### (a) Types of evidence utilized in UK marine decisions

**Figure 1.**
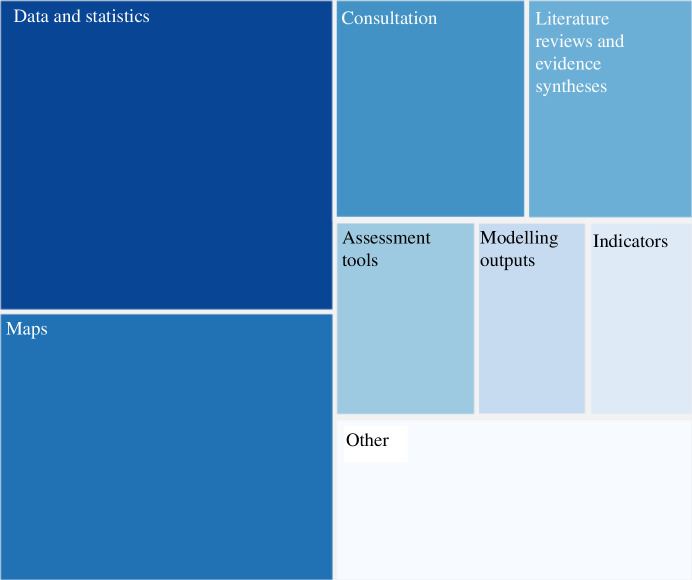
Relative proportions of references citing different types of evidence in the six case studies.

The results revealed various evidence types cited in the six marine case studies reviewed for this study (figure 1). Among these, the reviewed documents predominantly cited certain types of evidence, including quantitative and qualitative data and statistics, maps, consultations with stakeholders and the public, literature reviews and evidence syntheses, outputs of assessment tools and modelling exercises and indicators.

Conversely, the reviewed documents cited a variety of other evidence types less frequently. These included expert judgement, good practice guidance, photos providing visual documentation, feasibility studies, case studies, databases of evidence, environmental standards, government responses, interviews, questionnaires and workshops, all represented by the ‘other’ box in figure 1.

Table 1 presents the frequency and proportion of different types of evidence cited in the reviewed case studies across the Scottish, Welsh and English case studies. The results indicate a notable variation in the types of evidence utilized among the case studies from the three countries. For example, the Scottish case studies heavily relied on maps, with 48.0% of total references being maps, particularly in the Sectoral Marine Plan for Offshore Wind Energy [55]. The Welsh case studies cited a diverse range of evidence types, with significant reliance on consultation (20.8%) and other less frequently cited types of evidence (25.8%), such as case studies and expert judgement, reflecting a broad range of evidence types cited in the case study documents—especially in the Welsh Marine Evidence Report [62], which supported the WNMP. The English case studies showed a high dependence on data and statistics (34.9%) and literature reviews and evidence syntheses (13.8%), especially in the Southsea Coastal Defence Scheme.

**Table 1.** Frequency of evidence types cited in the case studies, measured by the number of references coded, which reflects the unique sections of text coded with a specific evidence type. The results are aggregated for Scotland, Wales and England by summing the frequency of each evidence type across the two case studies reviewed for each country. The table also shows the proportion of total evidence represented by each evidence type. The ‘other’ category includes less frequently cited evidence types, such as case studies, expert judgement and photographs.

	Scotland			Wales			England		
evidence type	total (*n*, % of total)	Sectoral Marine Plan for Offshore Wind Energy (*n*)	Sullom Voe Harbour Masterplan (*n*)	total (*n*, % of total)	Welsh National Marine Plan (*n*)	River Wye and River Usk Rod and Line Byelaws (*n*)	total (*n*, % of total)	Southsea Coastal Defence Scheme (*n*)	Southsea Coastal Defence Scheme sub-frontage 4 (*n*)
assessment tools	6, 1.5	1	5	64, 13.3.	52	12	14, 7.4	14	0
consultation	26, 6.5	7	19	100, 20.8	84	16	37, 19.6	28	9
data and statistics	119, 29.8	88	31	93, 19.4	81	12	66, 34.9	65	1
indicators	30, 7.5	11	19	3, 0.6	3	0	3, 1.6	3	0
literature reviews and evidence syntheses	12, 3.0	12	0	55, 11.5	51	4	26, 13.8	25	1
maps	292, 48.0	164	28	24, 5.0	24	0	20, 10.6	20	0
modelling outputs	9, 2.3	9	0	17, 3.5	15	2	18, 9.5	15	3
other	6, 1.5	0	6	124, 25.8	55	69	5, 2.6	5	0
total	400			480			189		

### (b) Evidence on natural capital and ecosystem services

#### (i) Evidence about natural assets

We identified various natural assets and the cited evidence for each. It is important to remember that natural assets represent the standing stock of nature before they are utilized or enjoyed in a way that benefits humans.

1. **Aquatic resources**: Various evidence types were found for aquatic resources, including fish stock assessments, which provide quantitative measures of fish populations. Indicators on contaminant levels in fish were also noted, which measure the condition of fish stocks. Additionally, maps of commercial fish spawning grounds were commonly referenced, illustrating these resources’ geographical distribution.
2. **Marine ecosystem**: the extent and ecological characteristics, which can be proxies of the condition of the asset, tended to be well evidenced, typically supported by ecological survey data. This evidence tended to be reported in HRAs and Environmental Statements, which are both legally required for developments. These tended to be lengthy documents, detailing various sources of highly technical survey data, modelling outputs and maps.
3. **Water resources**: evidence related to water resources primarily consisted of water quality assessments. These assessments provide crucial information on the status and trends of water conditions. Indicators of water pollution were also cited in the Scottish case studies, reflecting the impact of human activities on water quality.
4. **Wind resources**: for wind resources, the evidence included data on wind speeds and energy generation potential.
5. **Other natural assets**: the documents also covered evidence of other natural assets, including aggregate resources, oil and gas, wave and tidal energy resources. These assets were represented through various types of data, assessments and maps, highlighting their availability, distribution and potential for exploitation.

### (ii) Evidence about ecosystem services

In the reviewed documents, there was a significant focus on the benefits that humans derive from marine natural assets, categorized as ecosystem service benefits. These benefits encompass a range of ecosystem services, including:

— **Provisioning services:** the documents frequently cited economic data and statistics related to provisioning services. This included income and employment figures from fishing, reflecting the direct benefits of aquatic resources. Additionally, the Gross Value Added to the national economy from oil and gas extraction was referenced in the Scottish case studies.
— **Regulation and maintenance services:** these services were evidenced through various data. For instance, estimates of employment in the emerging carbon capture and storage industry illustrated the potential of this sector in regulating atmospheric carbon. Moreover, the economic and human costs associated with coastal erosion and flooding were frequently discussed, underscoring the critical role of marine ecosystems in protecting coastal areas and communities.
— **Cultural services:** the evidence for cultural services was diverse. This included the social benefits of recreational angling, which contributes to individual well-being and supports local economies, and consultation responses about access to the coastal environment and preservation of marine heritage sites. Statistics reported the state of historic marine sites and indicators were cited on the percentage of adults who live within a 5 min walk of their local green or blue space. Statistics related tp the economic value of benefits included the annual expenditure on marine recreation activities, the economic value of marine tourism, maritime navigation and trade and marine biodiversity conservation.

#### (iii) Comparison of evidence types in marine natural assets and ecosystem services

The comparative analysis of evidence types cited for marine natural assets and ecosystem service categories reveals a dominance of certain evidence types in relation to different natural capital components (table 1 and table 2).

**Table 2.** The relative frequency of coding intersections between evidence types and marine natural assets and ecosystem services (ES). The cells indicate the relative frequency of coding intersections: Very low citation count (VL), low (L), medium (M), high (H), and very high (VH).

	components of natural capital			
evidence types	natural assets	cultural ES	provisioning ES	regulation and maintenance ES
assessment tools	VH	L	H	VL
consultation	VH	VH	VH	L
data and statistics	VH	VH	VH	L
indicators	M	M	VL	VL
literature reviews and evidence syntheses	M	M	L	L
maps	VH	M	H	VL
modelling outputs	L	VL	VL	M

Assessment tools were cited most frequently to provide evidence on natural assets and ecosystem services. Examples of assessment tools include the Seascape Assessment of Wales, which assesses the character and special qualities of the Welsh Seascape [62,72], assessment tools for fisheries such as a species-specific tool for sea trout [73] and a climate change risk assessment for fisheries [74], an economic assessment of recreational uses related to marine biodiversity [75] and a Hydrodynamic and Sediment Assessment in the Environmental Statement [76] for the Southsea Coastal Defence Scheme.

Consultations were cited highly on natural assets, cultural and provisioning ecosystem services and to a lesser extent on regulation and maintenance of ecosystem services. Examples include the mapping of squid fishing areas by participants of public engagement events for the Sullom Voe Harbour Masterplan [51], a consultation response from the Shetland Fishermen’s Association detailing the areas of importance for juvenile commercial fish stocks [77], concerns expressed about access to the coastal environment and preservation of marine heritage sites during consultation events for the Southsea Coastal Defence Scheme [78] and a statutory consultee response about the importance of Priority Marine Features ‘not just for stabilizing the seabed. In some cases they are also important sinks for blue carbon’ [77].

Data and statistics were also cited highly for most components of natural capital. In terms of natural assets, examples include fish surveys that inform stock assessments [79] and ecological surveys that provide information on the status of marine biodiversity [80]. In terms of ecosystem services, examples include data on the spatial distribution of recreational sailing [81] and survey information on the characteristics of tourists visiting Portsmouth [82], Vessel Monitoring System data that record fish landings [83] and hydrological data from wave flow instruments to understand coastal erosion rates [84].

Indicators were most frequently cited to provide evidence on natural assets and cultural ecosystem services. Examples of indicators cited include the number of water bodies achieving ‘Good Ecological Status’ [85] as a measure of the condition of the marine ecosystem, the percentage of adults who live within a 5 min walk of their local green or blue space [83] as a measure of access to nature and the number of developments subject to flooding [85] as a measure of the natural disaster risk.

Literature reviews and evidence syntheses were most frequently cited to provide evidence on natural assets and cultural ecosystem services. Examples include a review of climate change impacts on fisheries [86] cited in the Rivers Wye and Usk Byelaw, a review of evidence on marine microplastic pollution [87] cited in Wales’ Marine Evidence Report, an evidence synthesis on catch-and-release recreational fishing [67] and literature reviews within the reviewed documents such as on the ecological disturbance caused by recreation in the HRA for the Southsea Coastal Defence Scheme [88] and on the ecosystem service benefits provided by kelp forests in Wales’ Marine Evidence Report [62].

Maps were cited as evidence for a range of natural capital components including minerals in the Welsh waters and Irish sea [89], marine habitat maps [90], maps showing the locations of marine recreation activities [51,62], historic maps used in a Heritage Assessment [91], maps of oil- and gas-related infrastructure [51], flood zone maps [52], sediment transport pathway maps [90] and coastal erosion risk maps [62].

Modelling outputs were most frequently cited to provide evidence on the regulation and maintenance of ecosystem services. Outputs from modelling included climate change scenario modelling to investigate future risks of erosion and flooding events [62,92,93] and modelling of the dynamics of marine pollution discharges [94], conservation limits derived from modelled fish stocks [95], spatial predictions of the occurrence of marine habitats [83] and fish spawning sites [96].

### (c) Gaps in the natural capital evidence

The gap analysis of ecosystem services in the six marine case studies reveals areas with robust evidence and notable gaps (table 3). In Provisioning Services, there was substantial evidence for biomass, mainly from fishing and aquaculture for food. Water use for energy generation and mineral and wind resources was well-documented, indicating a strong focus on these natural resources. Specific ecosystem services were frequently evidenced, like fishing and aquaculture in the Sullom Voe Harbour Masterplan case study, and extracting aggregates, oil and gas in the WNMP. Conversely, using wild and cultivated aquatic plants for food, materials and energy was less frequently documented.

**Table 3.** Assessment of Ecosystem Services Evidence in UK Marine Decisions. The table follows the CICES and GOAP classification systems, categorizing them into provisioning (orange), regulating and maintenance (green) and cultural (blue) ecosystem services. It reports the frequency of evidence in the reviewed documents for each ecosystem service by country. For each service and country, the proportion of evidence from the two case studies is presented as percentages. The ‘none’ category indicates no evidence identified for that ecosystem service.

section	division	group	examples	Scotland (offshore wind, Sullom Voe)	Wales (WNMP, Wye/Usk)	England (Southsea, sub-frontage 4)
provisioning (biotic)	biomass	cultivated aquatic plants for nutrition, materials or energy	aquaculture production for food; cultivation of seaweed and kelp for energy	6 (16.7%, 83.3%)	3 (100%, 0%)	0 (0%, 0%)
		reared aquatic animals for nutrition, materials or energy	aquaculture production for food	46 (26.1%, 73.9%)	16 (100%, 0%)	5 (100%, 0%)
		wild plants (terrestrial and aquatic) for nutrition, materials or energy	fish, seaweed and kelp harvested for food and energy	2 (0%, 100%)	4 (100%, 0%)	0 (0%, 0%)
		wild animals (terrestrial and aquatic) for nutrition, materials or energy	fish and shellfish harvested for food	90 (13.3%, 86.7%)	67 (61.2%, 38.8%)	11 (100%, 0%)
provisioning (abiotic)	water	surface water used for nutrition, materials or energy	tidal and wave power for energy; water for domestic and industrial use	10 (0%, 0%)	26 (92.3%, 7.7%)	2 (100%, 0%)
	non-aqueous natural abiotic ecosystem outputs	mineral substances used for nutrition, materials or energy	aggregates and minerals extracted from seabed; oil and gas extracted for energy	14 (64.3%, 35.7%)	65 (98.5%, 1.5%)	0 (0%, 0%)
		non-mineral substances or ecosystem properties used for nutrition, materials or energy	wind used for energy	22 (0%, 0%)	22 (100%, 0%)	0 (0%, 0%)
regulation and maintenance (biotic and abiotic)	transformation of biochemical or physical inputs to ecosystems	mediation of wastes or toxic substances of anthropogenic origin by living processes	carbon sequestration and storage by marine environment; disposal of wastes in the marine environment	10 (70%, 30%)	10 (100%, 0%)	0 (0%, 0%)
	regulation of physical, chemical and biological conditions	regulation of baseline flows and extreme events	marine/coastal features that control erosion; kelp reducing movement of intertidal sediment; coastline acting as sea defence, reducing flooding risk	7 (42.9%, 57.1%)	24 (100%, 0%)	31 (90.3%, 9.7%)
		lifecycle maintenance, habitat and gene pool protection	habitat and nursery ground provision for aquatic species	56 (44.6%, 55.4%)	33 (97%, 3%)	36 (100%, 0%)
		regulation of substrate quality	none	0 (0%, 0%)	0 (0%, 0%)	0 (0%, 0%)
		water conditions	none	0 (0%, 0%)	0 (0%, 0%)	0 (0%, 0%)
		atmospheric composition and conditions	none	0 (0%, 0%)	0 (0%, 0%)	0 (0%, 0%)
cultural (biotic and abiotic)	direct, *in situ* and outdoor interactions with living systems that depend on presence in the environmental setting	physical and experiential interactions with natural environment	recreation and tourism activities, e.g. walking, fishing, swimming; access to nature; visual amenity; health and well-bring impacts	46 (50%, 50%)	87 (92%, 8%)	104 (98.1%, 1.9%)
		intellectual and representative interactions with natural environment	marine and coastal heritage assets and activities, e.g. traditional fishing methods; land/seascapes appreciated for their unique characteristics	72 (25%, 75%)	38 (81.6%, 18.4%)	54 (96.3%, 3.7%)
	indirect, remote, often indoor interactions with living systems that do not require presence in the environmental setting	spiritual, symbolic and other interactions with natural environment	coastal and marine scapes of value	3 (0%, 100%)	5 (100%, 0%)	0 (0%, 0%)

The most cited ecosystem services for regulation and maintenance services were habitats and nursery grounds for aquatic species, highlighting their role in maintaining fish populations essential for fishing activities. The regulation of erosion and flood mitigation was also frequently evidenced, particularly in the WNMP and the Southsea Coastal Defence Scheme. However, significant gaps were noted in the evidence related to natural processes maintaining water quality, substrate quality regulation and atmospheric and oceanic conditions.

Cultural services were well represented in direct interactions with the marine environment, such as recreation, tourism and aesthetic enjoyment. These services were most frequently evidenced in the Southsea Coastal Defence Scheme case study, where substantial evidence was gathered through consultations about direct human use of the coastline. Marine heritage, including the conservation of heritage assets, was also frequently evidenced.

The documents where evidence on cultural ecosystem services might be expected to be reported, such as social impact assessments (SIA), equality impact assessments and sustainability appraisals, were available for several decisions. These documents primarily contained evidence about direct cultural ecosystem services like access to nature, recreation and heritage. Indirect cultural ecosystem services were more frequently reported in other documents:

— Welsh Marine Evidence Report: cited a valuation study on the spiritual and cultural well-being benefits of biodiversity [66].
— WNMP Sustainability Appraisal: an addendum highlighted the strong coastal links to Welsh culture and identity [97].
— Strategic Scoping Exercise for the WNMP: noted the intrinsic importance of Welsh seas for their habitats, seascapes, beauty, heritage and ‘sense of place’ [72].
— Sullom Voe Harbour Masterplan: described the spiritual enrichment, cognitive development and reflection provided by coastal connections. The accompanying Environment Report emphasized the psychological and spiritual benefits of wild areas in Shetland [51].

These findings indicate that while direct-use cultural ecosystem services are well-represented in SIAs and related documents, non-use values such as spiritual and symbolic connections are less frequently cited but still present in some assessments.

When comparing the weighting of evidence across the three countries and different case studies, disparities become evident. The evidence cited in the Scottish case studies strongly emphasized natural assets like aquatic resources and culturally important marine scapes. The evidence cited in the Welsh case studies focused more on aggregate and mineral resources, while in the English case studies, the evidence was predominantly about physical and experiential interactions with the marine and coastal environment. These differences are likely owing to the contrasting foci of the case studies reviewed, rather than national priorities.

### (d) Monetary valuation in the documents

The documents contained monetary valuations across various natural capital components, particularly highlighting the economic value of ecosystem services. For Provisioning Ecosystem Services, this encompassed tangible financial contributions from industries such as aggregates (critical for construction), aquatic resources (generating income through fishing and aquaculture), oil and gas (contributing significantly to national economies) and the growing renewable energy sector. We encountered less evidence for Regulation and Maintenance Services, and this evidence was focused solely on the financial burden of property damage owing to coastal flooding. In Cultural Ecosystem Services, valuation evidence was limited to the economic value of marine recreation and tourism industries and a valuation of the non-use values of marine biodiversity in Wales’ Marine Evidence Report.

## 4. Discussion

Our study illustrates how marine decisions in the UK are informed by evidence and the extent to which they consider the spectrum of marine natural capital and ecosystem services. We uncover a dominant reliance on specific evidence types, such as data and statistics, consultations, literature review and evidence syntheses, assessment tools, maps, modelling outputs and indicators, through a detailed analysis of six marine case studies across England, Scotland and Wales. This study underscores significant gaps in evidence for critical areas like water regulation and maintenance services provided by marine habitats, highlighting the necessity of bringing existing evidence on this ecosystem service into marine decisions and for investment in filling evidence gaps. Certain cultural ecosystem service benefits, including recreation and tourism, were well evidenced, but other less direct cultural ecosystem service benefits, such as spiritual and symbolic experiences, were not. By elucidating these gaps, our findings offer crucial insights for enhancing marine management strategies in the UK, stressing the importance of a more inclusive understanding and representation of marine ecosystem services to foster sustainable and holistic marine ecosystem management.

### (a) What types of evidence are utilized in UK marine decisions?

Our research identified a significant reliance on science-based evidence in UK marine decisions, a practice in line with DEFRA's and the MMO’s definitions of evidence. DEFRA’s broad definition of evidence, which the MMO aligns with, encompasses various scientific disciplines and methodologies, mirroring the dominant evidence types identified in our research: *‘Evidence is information used to support decisions… Evidence covers a range of sciences including economics, social research, operational research, statistics, natural science, engineering and geography. It includes research and development, monitoring and surveillance, and secondary analysis and synthesis*’ [98, p. 12]. This reflects a strong, science-based emphasis in England’s marine management, endorsing quantifiable and objective evidence. However, this prioritization may exclude non-scientific evidence types, such as anecdotal evidence from stakeholders with local experiences and opinions. For example, in the Rivers Wye and Usk Byelaw case study, stakeholder submissions were considered less objective and credible compared to the scientific fishery stock assessment that informed the byelaw. The Inquiry Report describes the consultee evidence as follows, *‘There has been a sorry dearth of objective scientific evidence put forward by objectors… The vast bulk of objectors’ cases has been simply the assertion of opinion, and anecdotal evidence*’ [56, p. 8]. Indeed, in other fields, the evidence-based movement that prioritizes scientific evidence has been criticized as being exclusionary of other forms of knowledge [99]. This prioritization of scientific evidence potentially overlooks other valuable insights, highlighting a discrepancy between the highly valued scientific evidence and the broader knowledge spectrum necessary for a comprehensive understanding of marine management.

The results also revealed notable variations in the types of evidence utilized among the Scottish, Welsh and English case studies. The Scottish case studies heavily relied on maps, particularly in the Sectoral Marine Plan for Offshore Wind Energy. The Welsh case studies cited a diverse use of evidence types, especially in the WNMP. The English case studies depended predominantly on data, statistics and literature reviews, particularly in the Southsea Coastal Defence Scheme. These differences illustrate the diverse evidence landscapes influencing policy and planning in each context. Furthermore, the frequent use of assessment tools, maps and consultations indicates a reliance on visual and participatory evidence forms, which can help in understanding spatial and stakeholder dimensions of marine issues.

### (b) How representative is this evidence of different marine natural capital and ecosystem services values?

Despite the case studies pre-dating the integration of the natural capital concept into decision-making processes, our findings show a reasonably comprehensive coverage of the range of ecosystem services. This indicates a broader, albeit implicit, recognition of the various values associated with marine natural capital and ecosystem services within these case studies. While rooted in traditional science-based perspectives, the evidence spans a spectrum of ecosystem services. This suggests an inherent acknowledgement of marine environments’ multifaceted benefits, even before the formal adoption of the natural capital concept in marine management and decision-making.

However, the evidence was dominated by particular assets and ecosystem services, particularly on fisheries, which aligns with the findings of [100] that ‘*fisheries, are the most well understood aspect of the natural capital benefits of the marine environment*’. In addition, while a diversity of evidence sources was typically cited on the state of natural assets in statutory documents like HRAs and Environmental Statements, it is not clear how appropriate this information is for understanding the condition of marine habitats and their ability to sustain the delivery of ecosystem service benefits. There is a risk that the evidence reported is simply the data that are easy to collect [101] rather than what is needed to understand the relationships between asset condition to ecosystem service benefits Identifying these gaps and strategically filling them is a priority [102].

DEFRA has identified cultural ecosystem service benefits as ‘*the most significant data gaps for application of the natural capital approach to the marine environment*’ [100]. However, we found a significant amount of evidence on cultural ecosystem services, reported by a range of evidence types, and not just limited to consultation responses as might be expected. However, the evidence was predominantly about recreation and tourism, potentially because they are the most well-studied marine and coastal cultural ecosystem services [17]. Both are economically valuable marine industries and can be evidenced by economic data on visitor numbers and spending. Such data do not tell you about the other non-economic benefits of human interaction with the marine environment, such as health and well-being. Indeed Mulholland and colleagues [100], identified a ‘*detailed understanding on the extent, nature and full set of benefits related to recreational activities*’ as a priority evidence gap to fill.

### (c) Are there any significant gaps in the representation of natural capital and ecosystem service values in the evidence base?

Evidence on the value of ecosystem services was most commonly cited for provisioning ecosystem services, such as from sales of fish and fossil fuels, and on direct cultural ecosystem services of recreation and tourism. There was less valuation evidence on regulation and maintenance services, such as water quality regulation, carbon sequestration and disaster mitigation. This finding aligns with previous evidence from reviews of the UK's natural capital [103], which identified existing evidence gaps as including ‘*regulating services including mediation of waste, toxins and nuisances; lifecycle, habitat and gene pool protection; and regulation of the chemical condition of the atmosphere and ocean. There is also a lack of values for certain cultural services, particularly marine and maritime heritage, spiritual and inspirational interactions, and health and well-being*’.

Our review reveals a significant gap in representing the ecosystem service of water quality maintenance. Various marine habitats, including littoral and sublittoral sediments, saltmarshes and reedbeds, oyster reefs, coastal saltmarsh and seagrass meadows, are instrumental in delivering valuable water quality regulation and carbon sequestration services in the UK [104]. However, these ecosystem services were rarely evidenced in the reviewed case studies, a concerning omission considering the importance of bathing water quality in some of the decisions that we reviewed. Water quality maintenance is one of the most frequently studied marine and coastal ecosystem services [17]. It is imperative that this information is evidenced in decisions so that decision-makers can gauge the extent of service loss when these critical habitats are degraded or lost owing to a decision. Indeed, Britain has lost most of its native oyster beds owing to overexploitation [105] and loss and threats to these habitats are ‘*largely overlooked*’ [106].

### (d) Implications for marine decision-making

The lack of evidence on marine habitats' water maintenance and regulation services could lead to decisions that fail to consider the full implications of the alteration of relevant habitats, whose degradation has substantial ecological and economic consequences [106,107]. Filling this gap requires investment in studies on the relationship between marine habitat functions and delivery of water quality improvement benefits, along with initiatives to bring such evidence into decision-making. A narrow focus on quantifiable scientific evidence may underrepresent indirect cultural services, such as symbolic and spiritual values, leading to decisions that fail to consider the full spectrum of human–marine relationships. Addressing these gaps requires greater use of the social sciences [100] as well as approaches from a broader spectrum of disciplines such as the arts and humanities [108].

Our findings are particularly relevant to emerging trends in UK environmental management. Organizations such as DEFRA, NatureScot and Natural Resources Wales have acknowledged evidence gaps concerning the benefits that nature provides to people. DEFRA is actively working to fill these gaps, notably through its Marine Natural Capital Evidence Assessment (mNCEA) programme, focusing on commissioning evidence production on marine ecosystem service benefits and the state of marine natural assets [22]. Similarly, NatureScot has highlighted the need for strategic evidence on nature’s benefits [48], while Natural Resources Wales has prioritized understanding specific marine ecosystem services like carbon sequestration and flood risk mitigation [49]. These priorities underline a national move towards a more comprehensive and informed approach to marine environmental management and decision-making.

### (e) Limitations

Natural capital is valued for its goods and services [109], highlighting that ecosystems offer diverse benefits beyond those with direct economic value. However, by adopting a natural capital perspective we may have overlooked values that are not well represented by these frameworks, including non-use values and values of marginalized or indigenous communities [110]—for example, the sense of identity experienced by individuals living within a fishing community or the inspiration evoked by gazing at the sea’s shimmering surface. These nuanced values may require alternative frameworks for comprehensive exploration. The Intergovernmental Science-Policy Platform on Biodiversity and Ecosystem Services highlights the importance of acknowledging various ways of understanding and valuing nature [111,112], indicating that other ways of valuing nature might reveal different aspects or gaps not captured by a natural capital approach.

The gaps we identified in the evidence of ecosystem services may not necessarily indicate a universal shortfall but rather reflect the specific focus and relevance of the issues to the six case studies we reviewed. Consequently, these case studies might not fully encapsulate the diversity of scenarios where natural capital concepts are applied, potentially skewing our assessment of evidence representation across a broader range of environmental management contexts. Additionally, we did not delve into the values embedded within the underlying institutions and decision-making processes related to marine management in the UK. By solely examining the cited evidence, we missed understanding the impact that evidence had on the actual decisions made.

Our analysis was based on the language used in the documents, which may have obscured some evidence characteristics. For instance, the distinction between tourism and recreation ecosystem services can be unclear. Recreational activities like sailing and coastal walking, enjoyed by locals and tourists, can be classified as recreation or tourism, depending on the reported data. However, since our study does not compare residents’ and tourists’ benefits, this lack of granularity does not impact our study’s conclusions.

Natural capital approaches illuminate the interconnected nature of the state of natural assets and the flows of benefits they provide. A strength of this approach is that declines in benefits can be attributed to environmental declines. However, in this study, we did not assess whether evidence on the state of natural assets was connected to evidence on the delivery of ecosystem service benefits in our six case studies. We did, however, notice that the evidence on natural assets and ecosystem services was typically presented in different documents, and the connection between them was not clearly explained. If the full potential of the natural capital concept is to be realized by marine regulators, it is crucial that the connection between the state of the marine environment and its capacity to sustain the delivery of benefits over time be made explicit. Indeed, the mNCEA has already identified this evidence gap and stated: ‘*there are significant gaps in understanding how habitats and species support the delivery of ecosystem services*’ [102,103].

## 5. Conclusions

Our study highlights significant insights and identifies gaps within the context of marine environmental management in the UK, utilizing a natural capital approach. Despite the broad coverage of ecosystem services in the case studies pre-dating the formal adoption of this approach, we identified areas of underrepresentation, particularly concerning the regulation of water quality by marine habitats and indirect human interactions with marine and coastal environments. UK marine management organizations and regulators are actively working to address gaps in our understanding of the benefits that marine and coastal ecosystems provide. This study reinforces the importance of their efforts. It highlights the ongoing need to expand our evidence base, ensuring that marine management strategies in the UK are informed by a comprehensive understanding of the multiple benefits of marine natural capital, especially in ensuring it encompasses a broader array of ecological, social and economic values. This broadened perspective is crucial for the sustainable and equitable stewardship of marine resources.

## Data Availability

The data used in this research are available in publicly available online documents listed in full in the electronic supplementary material [113].
